# Cymensifin A: a promising pharmaceutical candidate to defeat lung cancer via cellular reactive oxygen species-mediated apoptosis

**DOI:** 10.3389/fphar.2024.1361085

**Published:** 2024-04-11

**Authors:** Bruno Cesar Costa Soares, Hnin Ei Ei Khine, Boonchoo Sritularak, Pithi Chanvorachote, Rosa Alduina, Rungroch Sungthong, Chatchai Chaotham

**Affiliations:** ^1^ Pharmaceutical Sciences and Technology Program, Faculty of Pharmaceutical Sciences, Chulalongkorn University, Bangkok, Thailand; ^2^ Department of Biochemistry and Microbiology, Faculty of Pharmaceutical Sciences, Chulalongkorn University, Bangkok, Thailand; ^3^ Department of Pharmacognosy and Pharmaceutical Botany, Faculty of Pharmaceutical Sciences, Chulalongkorn University, Bangkok, Thailand; ^4^ Center of Excellence in Natural Products for Ageing and Chronic Diseases, Faculty of Pharmaceutical Sciences, Chulalongkorn University, Bangkok, Thailand; ^5^ Department of Pharmacology and Physiology, Faculty of Pharmaceutical Sciences, Chulalongkorn University, Bangkok, Thailand; ^6^ Center of Excellence in Cancer Cell and Molecular Biology, Faculty of Pharmaceutical Sciences, Chulalongkorn University, Bangkok, Thailand; ^7^ Department of Biological, Chemical and Pharmaceutical Sciences and Technologies (STEBICEF), University of Palermo, Palermo, Italy

**Keywords:** lung cancer, ROS, apoptosis, mitochondrial outer membrane permeabilization, DNA damage, Cymensifin A, orchid

## Abstract

**Background:** The upgrade of natural products for cancer treatment is essential since current anticancer drugs still pose severe side effects. Cymensifin A (Cym A) isolated from an orchid Cymbidium ensifolium has shown its potential to induce the death of several cancer cells; however, its underlying molecular mechanisms are hitherto unknown.

**Methods:** Here, we conducted a set of *in vitro* preliminary tests to assess the cytotoxic effects of Cym A on non-small-cell lung cancer (NSCLC) cells (A549, H23, H292, and H460). A flow cytometry system and Western blot analyses were employed to unveil molecular mechanisms underlying cancer cell apoptosis caused by Cym A.

**Results:** Cym A at 25–50 μM caused the death of all NSCLC cells tested, and its cytotoxicity was comparable to cisplatin, a currently used anticancer drug. The compound induced apoptosis of all NSCLC cells in a dose-dependent manner (5–50 μM), proven by flow cytometry, but H460 cells showed more resistance compared to other cells tested. Cym A-treated H460 cells demonstrated increased reactive oxygen species (ROS) and downregulated antioxidants (catalase, superoxide dismutase, and thioredoxin). The compound also upregulated the tumor suppressor P53 and the pro-apoptotic protein BAX but downregulated pro-survival proteins (BCL-2 and MCL-1) and deactivated survival signals (AKT and ERK) in H460 cells. Cym A was proven to trigger cellular ROS formation, but P53 and BAX were 2-fold more activated by Cym A compared to those treated with hydrogen peroxide. Our findings also supported that Cym A exerted its roles in the downregulation of nuclear factor erythroid 2–related factor 2 (a regulator of cellular antioxidant activity) and the increased levels of cleaved poly (ADP-ribose) polymerase (PARP) and cleaved caspase 3/7 during apoptosis.

**Conclusion:** We propose that Cym A induces lung cancer cell death via ROS-mediated apoptosis, while the modulation of cellular ROS/antioxidant activity, the upregulation of P53 and BAX, the downregulation or deactivation of BCL-2, MCL-1, AKT, and ERK, and the increased cleavage of PARP and caspase 3/7, were the elucidated underlying molecular mechanisms of this phytochemical. The compound can be a promising candidate for future anticancer drug development.

## Graphical Abstract

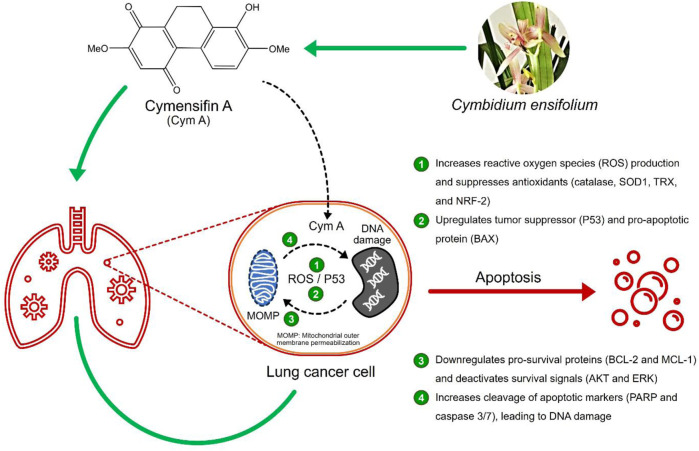

## 1 Introduction

Lung cancer is one of the most aggressive tumors and has a high prevalence and mortality rate, which contributes to the disease burden worldwide (Esposito et al., 2010; Kocarnik et al., 2022). Despite several decades of research and development of therapeutic approaches, the 5-year survival rate of lung cancer patients has not significantly improved (Lin et al., 2016). Chemotherapy, in combination with other treatments, is the recommended regimen prescribed for lung cancer patients at advanced stages (Patel et al., 2007; Crawford, 2013; Wood et al., 2018). Nevertheless, the suffering from chemotherapeutic side effects and the continuous growth of chemo-resistance, especially in clinical use, urge the search for a novel anticancer drug (Housman et al., 2014; Bukowski et al., 2020).

**FIGURE 1:**
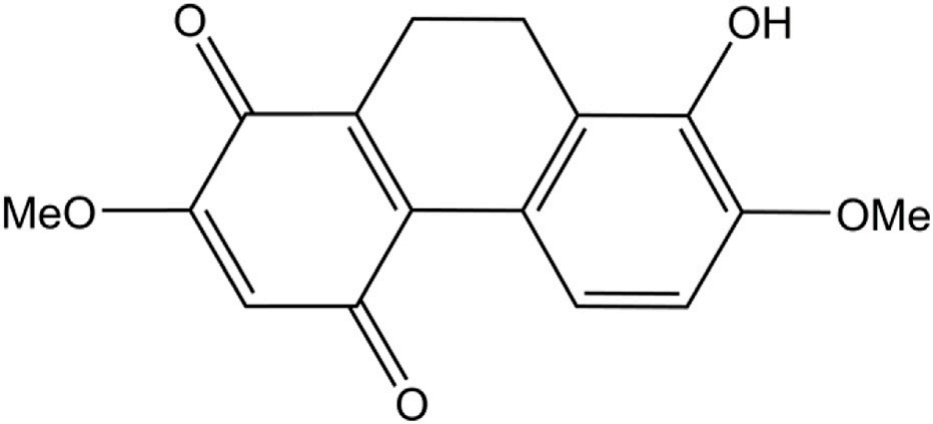
Chemical structure of cymensifin A isolated from *Cymbidium ensifolium*.

Various natural compounds have therapeutic potentials and low adverse effects on human health, and so are of interest in anticancer research (Nobili et al., 2009; Śliwiński et al., 2022). Several orchid species of the Orchidaceae family are among the promising sources to search for anticancer agents and have been shown for their cytotoxicity against different malignant cells, including those derived from human lung cancers (Jimoh et al., 2022a; Jimoh et al., 2022b; Śliwiński et al., 2022). Recently, we found that a dihydrophenanthrene, cymensifin A (Cym A) (8-hydroxy-2,7-dimethoxy-9,10-dihydrophenanthrene-1,4-dione) from the orchid *Cymbidium ensifolium* (Figure 1), causes cytotoxic effects on human lung cancer cells with a higher safety profile than cisplatin–the most widely used chemotherapeutic drug for cancer treatment (Jimoh et al., 2022a). However, the mechanisms underlying the anticancer activity of Cym A have yet been unknown.

Induced apoptosis of cancer cells is a pivotal mechanism to defeat cancer growth and metastasis. Apoptosis, a programmed cell death mediated by specific cellular signaling, is a focal mode of action in cancer chemotherapy (Strasser and Vaux, 2020). The imbalance between pro- and anti-apoptotic proteins leads to apoptosis dysregulation and cancer development (Scorrano and Korsmeyer, 2003). B-cell lymphoma 2 (BCL-2) and myeloid cell leukemia 1 (MCL-1), the pro-survival members of BCL-2 family proteins, are upregulated in lung cancer cells and play a critical role in evading apoptosis and drug resistance (Kluck et al., 1997; Thomas et al., 2010; Xiang et al., 2018). The expression of these pro-survival proteins and other proteins like BCL-2-associated X protein (BAX) (a pro-apoptotic protein) together with survival signals [e.g., protein kinase B (AKT) and extracellular signal-regulated kinase (ERK)], is mediated by the tumor protein P53 (Wei et al., 2001; Chipuk et al., 2004; Hemann and Lowe, 2006; Parrales and Iwakuma, 2015).

After accumulating damaged DNA or other cellular stresses, P53 downregulates BCL-2 and MCL-1 and upregulates BAX to execute apoptosis via the mitochondrial outer membrane permeabilization (MOMP) (Chipuk et al., 2004; Hemann and Lowe, 2006). Lung cancer cells generally escape apoptosis via BAX suppression and AKT/ERK overexpression (Viktorsson et al., 2005; Yang et al., 2018). P53 activation due to the response to reactive oxygen species (ROS) is another pathway inducing programmed cell death. Hydrogen peroxide (H_2_O_2_), a potent ROS, triggers canonical DNA damage, causing single- and double-strand DNA breaks that activate P53 (Vazquez et al., 2008). Some cellular defensive proteins, such as catalase, superoxide dismutase (SOD), thioredoxin (TRX), and nuclear factor erythroid 2–related factor 2 [NRF-2, a regulator in cellular antioxidant activity (Ma, 2013)], can overcome oxidative stress in cancer cells. All these proteins assist cancer cells in maintaining the ROS level that is unharmed and not reaching over the incompatible threshold with cell survival (Viktorsson et al., 2005).

DNA damage can be an upstream event of downstream MOMP and causes programmed cell death (Curtin and Szabo, 2020). Cytochrome *c* released from MOMP activates a series of caspases, activated caspase 3/7 further cleaves poly (ADP-ribose) polymerase (PARP), and cleaved PARP causes nuclear DNA fragmentation, leading to apoptosis. The contribution of PARP cleavage by core caspase 3/7 in apoptosis has been well-recognized for decades (Curtin and Szabo, 2020). PARP cleavage occurs between Asp124 and Gly215 and results in a catalytic domain (89 kD) of the carboxyl-terminal fragment plus a DNA-binding domain (24 kD) of the amino-terminal segment (Fu et al., 2023). These cleaved PARP segments do not only contribute to apoptosis but also DNA repair, genome stability, and oncogenesis (Fu et al., 2023; Tan, 2020). Hence, the increased levels of cleaved PARP and cleaved caspase 3/7 are among the biological markers in MOMP-mediated cell death.

In this study, we aimed to investigate the underlying mechanisms of Cym A in cancer cell apoptosis, targeting the ROS-mediated pathways involved in MOMP and DNA damage. The possibility of applying this natural product in lung cancer therapy was also addressed and discussed.

## 2 Methods and materials

### 2.1 Chemicals and reagents

Cym A (Figure 1) was extracted from the aerial parts of *C. ensifolium* as described previously (Nobili et al., 2009). Reagents for cell culture, including Roswell Park Memorial Institute (RPMI) medium, fetal bovine serum (FBS), l-glutamine, Dulbecco’s Modified Eagle Medium (DMEM), streptomycin/penicillin solution, phosphate-buffered saline (PBS) at pH 7.4, and 0.25% trypsin containing 0.53 mM EDTA, were obtained from Gibco (Gaithersburg, MA, United States). The radioimmunoprecipitation assay (RIPA) lysis buffer and SuperSignal^®^ West Pico Chemiluminescent Substrate were purchased from Thermo Scientific (Rockford, IL, United States). A protease inhibitor cocktail was received from Roche Applied Science (Indianapolis, IN, United States). The bicinchoninic acid (BCA) protein assay kit and nitrocellulose membranes were sourced from Pierce Biotechnology (Rockford, IL, United States) and Bio-Rad Laboratories (Hercules, CA, United States), respectively. Cisplatin, Hoechst 33342, propidium iodide (PI), Tris-HCl, NaCl, Tween 20, 2′,7′-dichlorofluorescein diacetate (DCFH2-DA), and *N*-acetylcysteine (NAC), were bought from Sigma Chemical, Inc. (St. Louis, MO, United States). Dimethyl sulfoxide (DMSO) was obtained from EMD Millipore Corporation (Billerica, MA, United States). The annexin V/PI apoptosis kit and 3-(4,5-dimethylthiazol-2-yl)-2,5-diphenyltetrazolium bromide (MTT) were acquired from Biotium (San Francisco, CA, United States) and Life Technologies (Eugene, OR, United States), respectively. Specific primary antibodies (catalog number provided) against MCL-1 (94296), BAX (5023), P53 (9282), BCL-2 (4223), AKT (4691), p-AKT (Ser473) (4060), ERK (4695), p-ERK (Thr202/Tyr204) (4370), caspase-3 and cleaved caspase-3 (14220), caspase-7 and cleaved caspase-7 (12827), PARP and cleaved PARP (9542), and β-ACTIN (9470) as well as horseradish peroxidase (HRP)-linked anti-rabbit (7074) and anti-mouse (7076) secondary antibodies, were procured from Cell Signaling Technologies, Inc. (Denver, MA, United States). Primary antibodies (catalog number provided) against catalase, SOD1, TRX, and smooth muscle actin (SMA) (ab179843, for four antibodies) and NRF-2 (ab137550), were ordered from Abcam (Cambridge, United Kingdom).

### 2.2 Cell cultures

Human lung cancer A549 (wild type *TP53* and mutant *KRAS*), H23 (mutant *TP53* and *KRAS*), H292 (wild type *TP53* and *KRAS*), and H460 (wild type *TP53* and *KRAS*) cells were bought from the American Type Culture Collection (ATCC; Manassas, VA, United States). A549 cells were cultured in DMEM, while H23, H292, and H460 cells were maintained in the RPMI medium. All culture media were supplemented with 10% FBS, 2 mmol L^−1^ L-glutamine, and 100 units mL^−1^ streptomycin/penicillin solution. Cells were grown at 37°C in a 5% CO_2_ atmosphere until they reached 70%–80% confluence before being used for the experiments.

### 2.3 Cytotoxicity assay

Cell viability was assessed via MTT colorimetric assay. Every lung cancer cell was treated with varying concentrations (0, 5, 25, and 50 μM) of Cym A for 24 h, while 50 μM of cisplatin served as a positive control. After the treatments, the medium was removed, and cells at an initial density of 1 × 10^4^ cells/well in a 96-well plate were subsequently incubated with 0.4 mg mL^−1^ MTT solution for 3 h at 37°C, protected from light. The supernatant was then removed, and 100 μL DMSO was added to each well to dissolve the purple formazan crystals. The intensity of the formazan product was measured with an absorbance at 570 nm wavelength (A_570_), using a microplate reader (Anthros, Durham, NC, United States). The relative cell viability (%) was calculated from the A_570_ ratio between the treated and untreated [with 0.5% (v/v) DMSO] cells.

Dead cells caused by exposure to varying concentrations of Cym A for 24 h were confirmed by a nuclear staining method. Cells (1 × 10^4^ cells/well in a 96-well plate) in the controls and the treatments with Cym A as formerly described were subjected to a nuclear staining assay using 10 μg mL^−1^ Hoechst 33342 and 5 μg mL^−1^ PI. After staining at 37°C for 30 min, cells were observed under a fluorescence microscope (Olympus IX51 with DP70, Olympus, Japan), where dead cells displayed bright blue Hoechst 33342 fluorescence (due to nuclear condensation and fragmentation in apoptotic cells) and red PI fluorescence (late apoptotic).

### 2.4 Modes of cell death detection

A flow cytometry system was also conducted to define modes of cell death. Every lung cancer cell line (2 × 10^5^ cells/well in a 6-well plate) was treated with varying concentrations (0, 5, 25, and 50 μM) of Cym A and a set of controls for 24 h as previously described. Cells were collected from the 6-well plate and centrifuged at 400 *g*, 4°C, for 5 min before staining with annexin V and PI, following the manufacturer’s instruction. The amount of living, apoptosis, and necrosis cells was quantified using Guava^®^ easyCyte™ Flow Cytometer and InCyte 3.3 software (EMD Millipore, St. Louis, MO, United States).

### 2.5 Evaluation of cellular ROS

To detect the level of cellular ROS, a chosen lung cancer cell line (2 × 10^5^ cells/well in a 6-well plate) was stained with a DCFH2-DA fluorescence probe at 4°C for 30 min. The stained cells were then either incubated with (0, 5, 25, and 50 μM) Cym A or 300 μM H_2_O_2_ (positive control) for 0–6 h. Cells pretreated with NAC for 30 min before staining with DCFH2-DA served as a negative control. After centrifugation at 400 *g*, 4°C, for 5 min, the fluorescence intensity of DCFH2-DA (representing the cellular ROS level) was measured in all treated and control cells by using Guava^®^ easyCyte™ Flow Cytometer and InCyte 3.3 software (EMD Millipore, St. Louis, MO, United States).

### 2.6 Western blot analysis

The expression of some proteins involved in the cellular response to ROS, tumor suppression, and apoptosis/survival of a chosen lung cancer cell line, was determined by Western blot analysis. The chosen lung cancer cell line (2 × 10^5^ cells/well in a 6-well plate) was treated with varying concentrations (0, 5, 25, and 50 μM) of Cym A or a set of controls for 24 h as previously described. The supernatant was removed, and cells were incubated at 4°C for 30 min with the RIPA lysis buffer supplemented with a freshly prepared protease inhibitor cocktail. Cells were scraped on ice, and the cell lysates were centrifuged at 8,000 *g*, 4°C, for 10 min to collect the clear supernatant. Then, the supernatant was evaluated for protein content using a BCA assay kit. Equal amounts of protein were separated into 10% (w/v) sodium dodecyl sulfate-polyacrylamide gel electrophoresis (SDS-PAGE) before being transferred onto 0.45 μm nitrocellulose membranes. Each membrane was blocked with 5% skim milk in TBST pH 7.4 (25 mM Tris-HCl, 125 mM NaCl, and 0.1% Tween 20) at ambient temperature for 45 min and subsequently immersed in a specific primary antibody-containing solution at 4°C overnight. Then, the membrane was washed thrice with TBST for 5 min each and stained with the HRP-secondary antibody for 2 h at ambient temperature. After staining, the signals of specific proteins were detected using SuperSignal^®^ West Pico Chemiluminescent Substrate and quantified using the ImageJ software (Version 1.53k).

### 2.7 Statistical analysis

Data obtained from three independent experiments were computed and presented as means ± standard deviations. The statistical analysis was performed in SPSS version 28 (IBM Corp., Armonk, NY, United States) using the one-way analysis of variance (ANOVA) followed by Tukey’s *post hoc* test. Any probability (*p*)-values under 0.05 are considered statistically significant.

## 3 Results

### 3.1 Cym A induces lung cancer cell apoptosis

**FIGURE 2:**
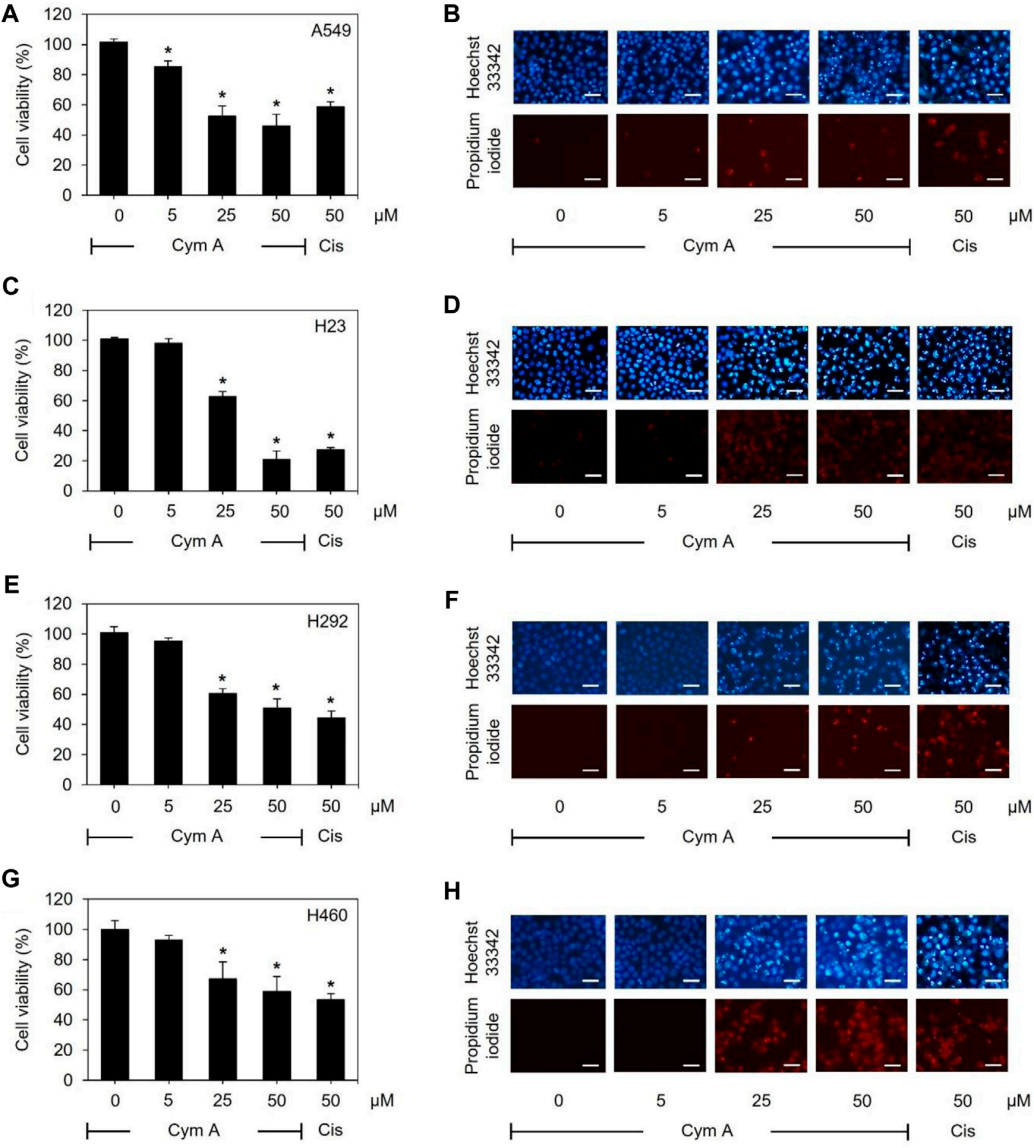
Cymensifin A (Cym A) poses cytotoxic effects on various lung cancer cells. A549 **(A,B)**, H23 **(C,D)**, H292 **(E,F)**, and H460 **(G,H)** cells were used in this experiment. Cells exposed to varying concentrations {0 [vehicle control, using 0.5% (v/v) dimethyl sulfoxide], 5, 25, or 50 μM} of Cym A or cisplatin (Cis) (positive control) for 24 h were assessed quantitatively by 3-(4,5-dimethylthiazol-2-yl)-2,5-diphenyltetrazolium bromide assay **(A,C,E,G)** and qualitatively by nuclear staining **(B,D,F,H)** for their viability. In nuclear staining assays, dead cells refer to those stained bright blue Hoechst 33342 fluorescence (nuclear condensation and fragmentation) and red propidium iodide fluorescence (late apoptosis), where scale bars are 100 μm. The numerical data represent means ± standard deviations derived from three independent experiments, where asterisks refer to the statistically significant difference of means compared to the untreated vehicle control (*p* < 0.05).

As assessed by MTT and nuclear staining assays (Figure 2), human lung cancer cells treated with varying concentrations of Cym A significantly affected their viability. Every lung cancer cell was susceptible to 25–50 μM Cym A, except for A549 cells that were sensible to 5 μM Cym A (Figure 2A). However, H460 cells (Figure 2G) showed a relatively higher degree of Cym A resistance when compared to other lung cancer cells tested (Figures 2A, C, E). Notably, 50 μM Cym A comparably decreased the relative cell viability of H460 cells to the same extent as 50 μM cisplatin (an approved anticancer drug as a positive control). Dead cells with condensed and fragmented nuclei stained with bright blue Hoechst 33342 and red PI fluorescence in a nuclear staining assay confirmed the cytotoxic effect of Cym A compared to cisplatin (Figures 2B, D, F, H).

**FIGURE 3:**
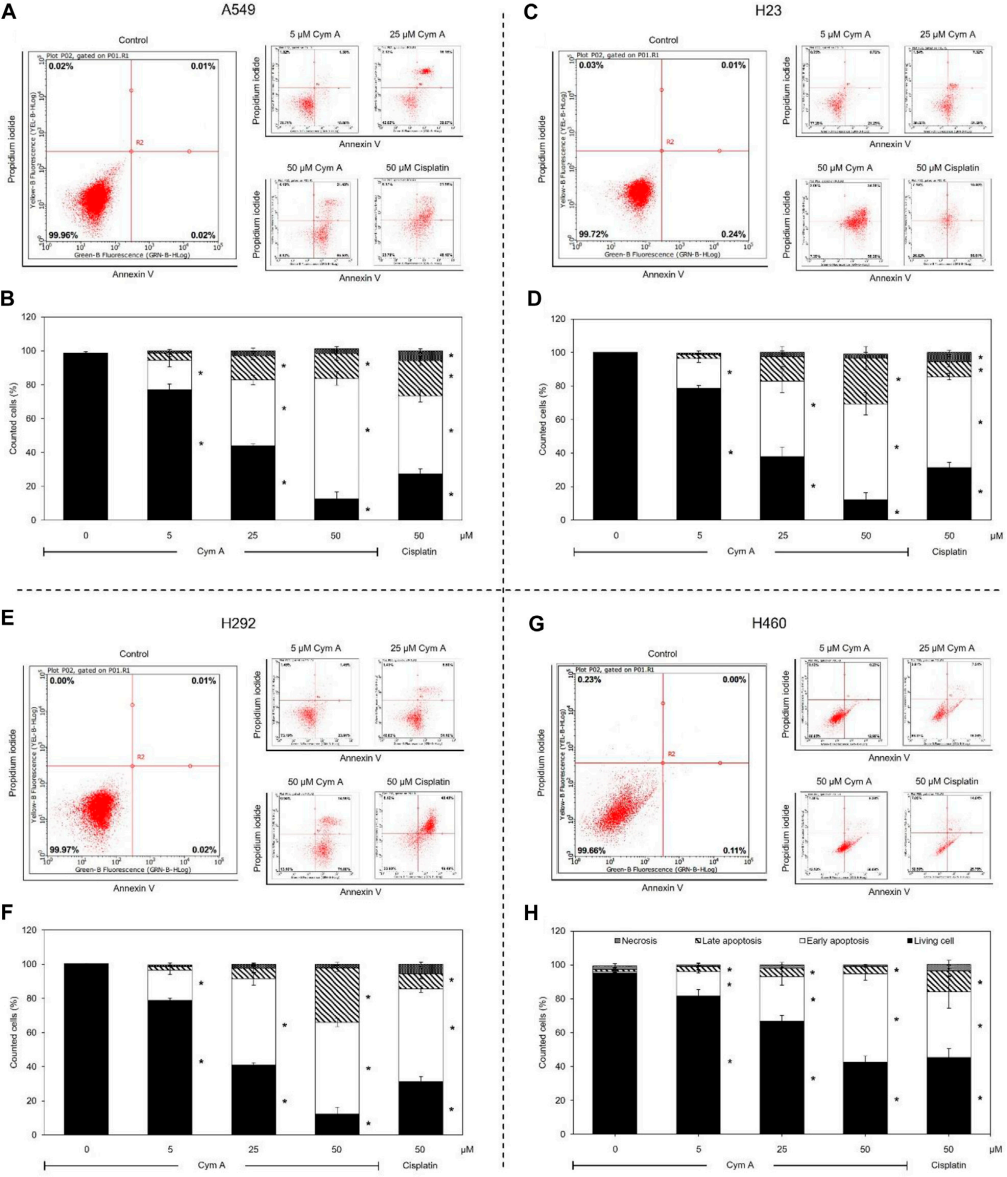
Cymensifin A (Cym A) causes lung cancer cell death via apoptosis. A549 **(A,B)**, H23 **(C,D)**, H292 **(E,F)**, and H460 **(G,H)** cells were used in this experiment. The mode of cell death assessed by an annexin V/propidium iodide staining-based flow cytometry system was conducted, which contributed to subpopulation-gating histograms as representatives chosen from three independent experiments **(A)** and a summary of every subpopulation (necrotic, late apoptotic, early apoptotic, and living cells) per each treatment **(B)**. Cells exposed to varying concentrations {0 [vehicle control, using 0.5% (v/v) DMSO], 5, 25, or 50 μM} of Cym A or cisplatin (positive control) for 24 h were analyzed. The numerical data represent means ± standard deviations derived from three independent experiments, where asterisks refer to the statistically significant difference of means compared to the untreated vehicle control (*p* < 0.05).

Modes of cell death after Cym A treatments were analyzed using an annexin V/PI staining-based flow cytometry system (Figure 3). Cym A showed similar cytotoxic effects on all lung cancer cells tested (Figures 3A–H) as observed with the MTT cell viability assay (Figures 2A, C, E, G). However, Cym A caused a significant reduction of H460 viable cells when treated with its lowest concentration (5 μM) (Figures 3G, H), which was not distinguishable by the MTT assay (Figure 2G). Either early (annexin V^+^/PI^−^) or late (annexin V^+^/PI^+^) apoptotic H460 cells increased significantly in dose-dependent manure, while necrotic H460 cells were unchanged (Figures 3G, H). This result also reversely correlated with the significant decrease of living H460 cells. Moreover, at the same concentration tested (50 μM), Cym A explicitly highly induced early apoptosis in H460 cells compared to cisplatin (a positive control) (Figure 3H).

### 3.2 Cym A induces ROS generation and downregulates antioxidants in lung cancer cells

**FIGURE 4:**
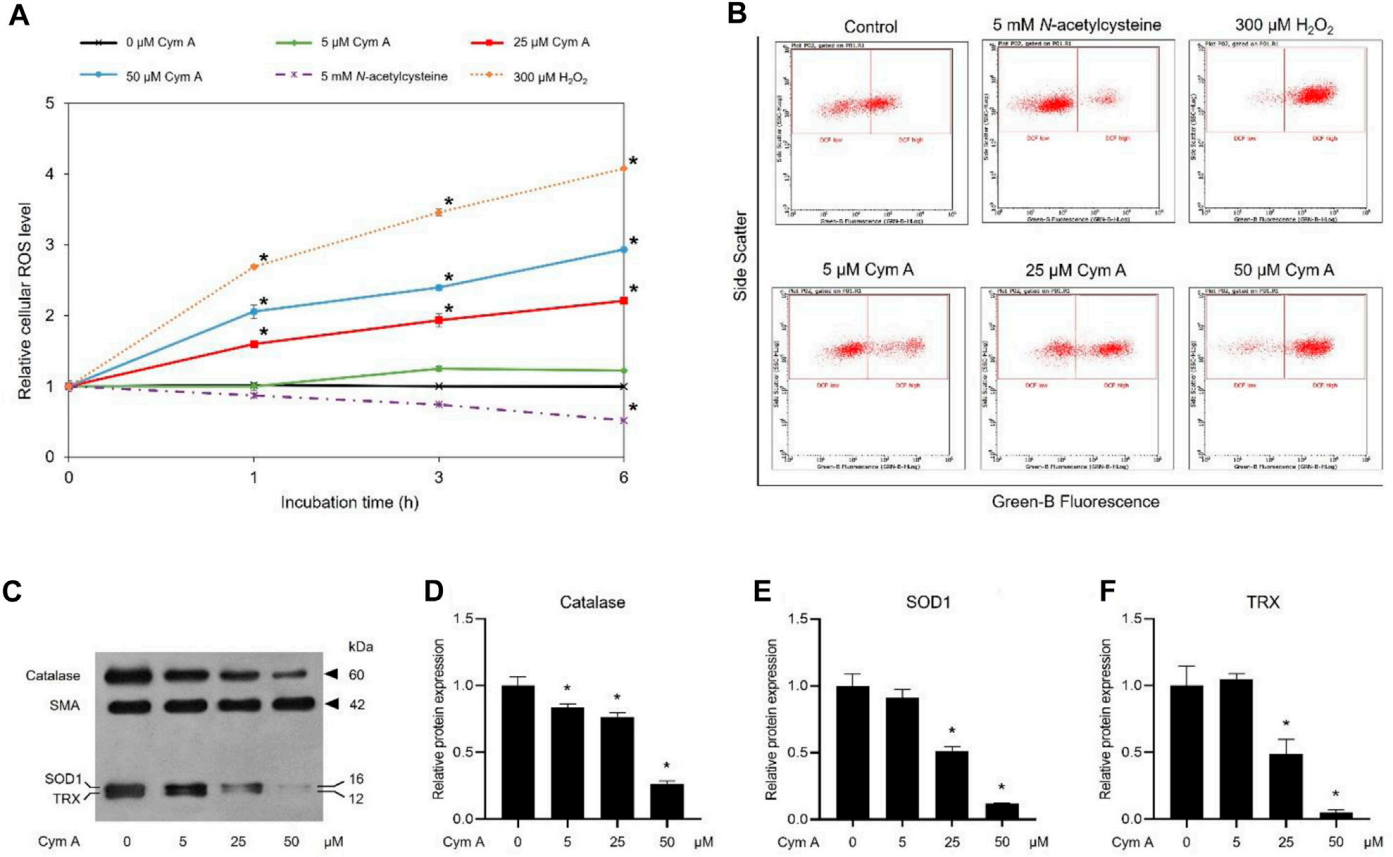
Cymensifin A (Cym A) causes reactive oxygen species (ROS) stress and downregulates antioxidants in lung cancer cells. H460 cells were used in this experiment. A 2′,7′-dichlorofluorescein diacetate (DCFH2-DA) fluorescence-probing flow cytometry assay was conducted to determine cellular ROS levels, which contributed to the relative value of ROS level at each measuring point to that derived from time zero **(A)** and subpopulation-gating histograms as representatives chosen from three independent experiments **(B)**. Cells exposed to varying concentrations {0 [vehicle control, using 0.5% (v/v) dimethyl sulfoxide], 5, 25, or 50 μM} of Cym A for up to 6 h were analyzed, while 300 μM hydrogen peroxide (H_2_O_2_)-treated cells served as positive controls. Cells pretreated with 5 mM *N*-acetylcysteine (negative control) were also included. The impact of Cym A on cellular antioxidant expression was assessed by a Western blot analysis (**C–F**, original blots can be found in Supplementary Material), where catalase **(C,D)**, superoxide dismutase 1 (SOD1) **(C,E)**, and thioredoxin (TRX) **(C,F)** were analyzed. The smooth muscle actin (SMA) served as a reference protein. The numerical data represent means ± standard deviations derived from three independent experiments, where asterisks refer to the statistically significant difference of means compared to the result at time zero **(A)** or the untreated vehicle control **(D–F)** (*p* < 0.05).

DCFH2-DA fluorescence-probing flow cytometry assay and Western blot analysis were conducted to prove how Cym A contributed to ROS formation and expression of cellular antioxidants in H460 cells (Figure 4). The ROS levels in H460 cells treated with 25 and 50 μM Cym A for up to 6 h significantly increased in a time-dependent manner (Figures 4A, B). Only Cym A at 25 and 50 μM exhibited a similar role in ROS generation to 300 μM H_2_O_2_ (a positive control) but different from untreated control and 5 mM NAC (a negative control)–a well-known antioxidant that suppresses ROS stress. A Western blot analysis also proved that Cym A-treated H460 cells have lower expression levels of cellular antioxidants, including catalase, SOD1, and TRX (Figures 4C–F).

### 3.3 Cym A upregulates tumor protein P53 and modulates apoptosis/survival-associated proteins

**FIGURE 5:**
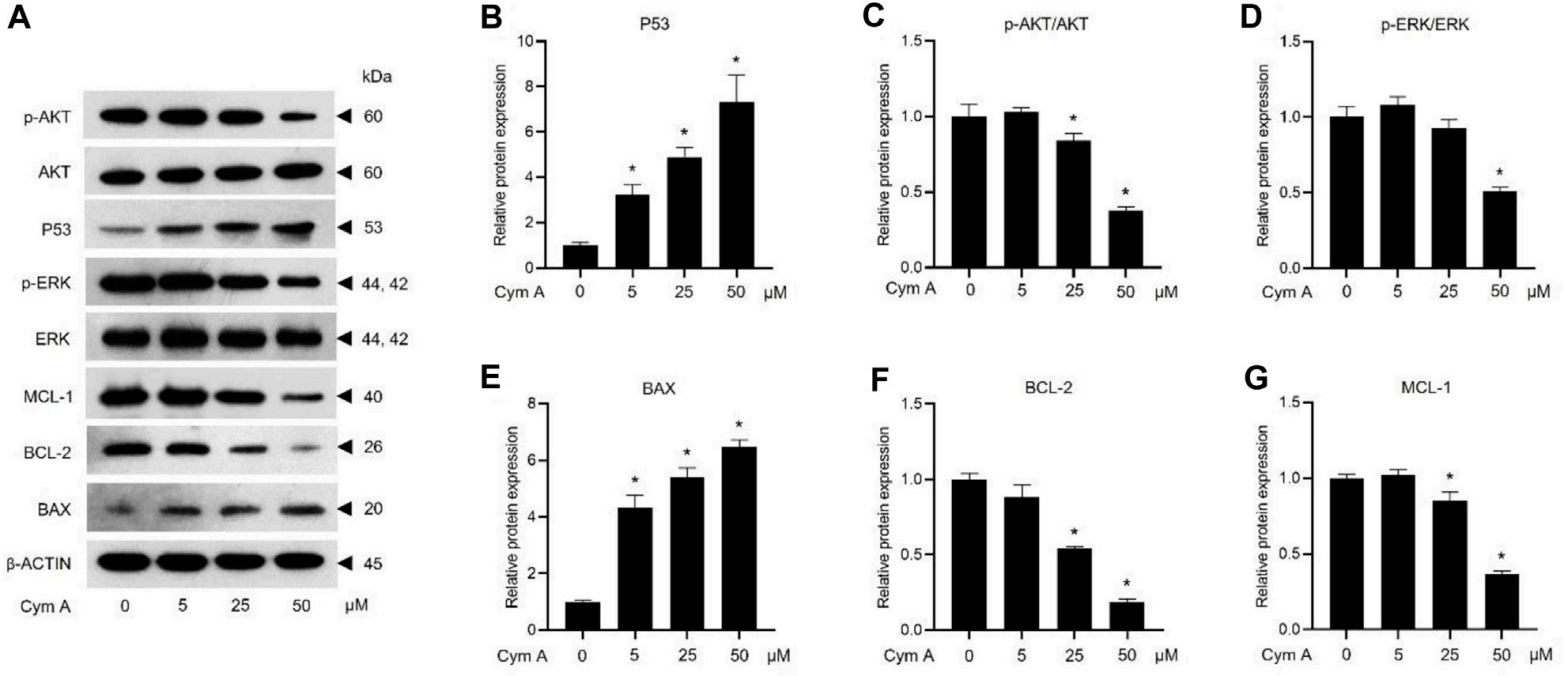
Cymensifin A (Cym A) upregulates tumor suppressor protein (P53) and modulates apoptosis/survival-associated proteins in lung cancer cells. H460 cells were used in this experiment. Cells exposed to varying concentrations {0 [vehicle control, using 0.5% (v/v) dimethyl sulfoxide], 5, 25, or 50 μM} of Cym A for 24 h were assessed by a Western blot analysis (**A–G**, original blots can be found in Supplementary Material), where P53 **(A,B)**, and other apoptosis/survival-associated proteins, including AKT **(A,C)**, ERK **(A,D)**, BAX **(A,E)**, BCL-2 **(A,F)**, and MCL-1 **(A,G)**, were analyzed (prefix p for AKT and ERK refers to their phosphorylated portion). The β-ACTIN served as a reference protein. The numerical data represent means ± SDs derived from three independent experiments, where asterisks refer to the statistically significant difference of means compared to the untreated vehicle control (*p* < 0.05).

The impacts of Cym A on the expression or activation of some proteins involved in tumor suppression (i.e., P53) and apoptosis/survival (i.e., BAX, BCL-2, MCL-1, AKT, and ERK)-associated markers in H460 cells, were evaluated by Western blot analysis (Figure 5). The compound significantly upregulated P53 (Figures 5A, B) and a pro-apoptotic protein, BAX (Figures 5A, E) in dose-dependent manure. Moreover, Cym A at 25–50 μM could downregulate pro-survival proteins, BCL-2 (Figures 5A, F) and MCL-1 (Figures 5A, G). Only at the relatively high concentrations (25–50 μM) of Cym A could significantly suppress the phosphorylation of survival-associated proteins, AKT (Figures 5A, C) and ERK (Figures 5A, D).

### 3.4 Cym A induces lung cancer cell death via ROS-mediated pathways

**FIGURE 6:**
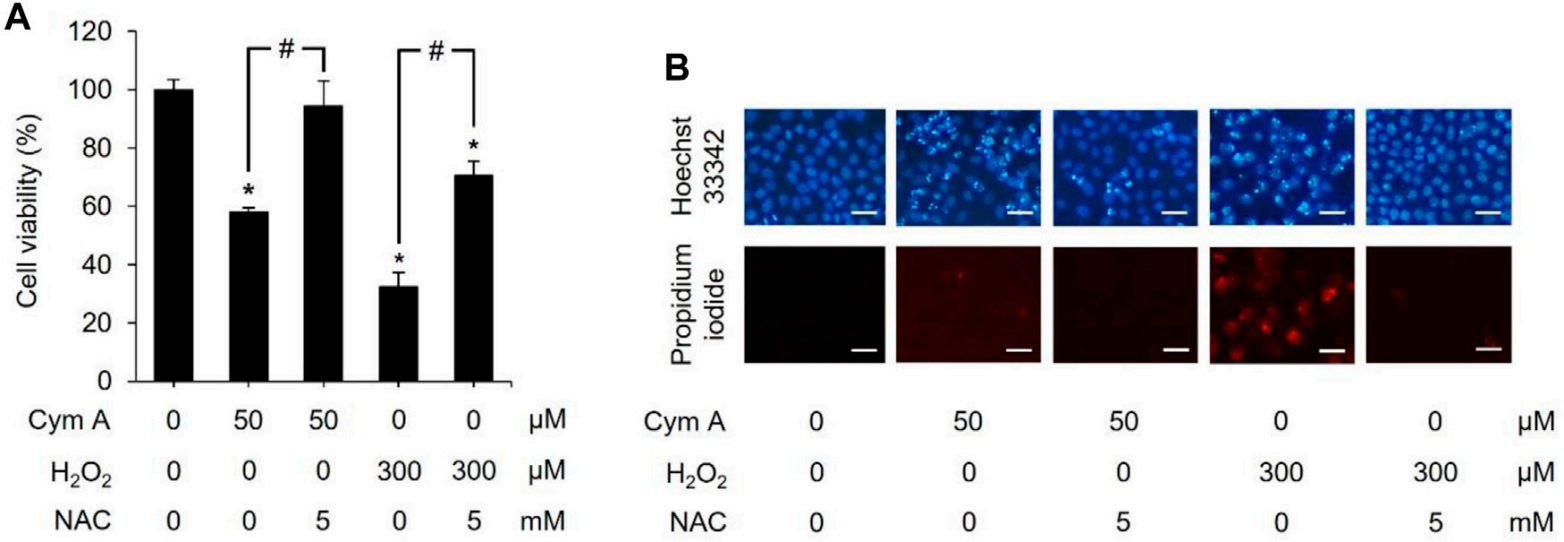
Cymensifin A (Cym A) acts as reactive oxygen species (ROS), and this role can be overcome by ROS inhibitor. H460 cells were used in this experiment. Cells exposed to 0 [vehicle control, using 0.5% (v/v) dimethyl sulfoxide] or 50 μM Cym A or 300 μM hydrogen peroxide (H_2_O_2_) (positive control) for 24 h were assessed quantitatively by 3-(4,5-dimethylthiazol-2-yl)-2,5-diphenyltetrazolium bromide assay **(A)** and qualitatively by nuclear staining **(B)** for their viability. Cells pretreated with 5 mM *N*-acetylcysteine (NAC) (ROS inhibitor) for 30 min before exposure to Cym A or H_2_O_2_ served as negative controls. In nuclear staining assays **(B)**, dead cells refer to those stained bright blue Hoechst 33342 fluorescence (nuclear condensation and fragmentation) and red propidium iodide fluorescence (late apoptosis), where scale bars are 100 μm. The numerical data represent means ± standard deviations derived from three independent experiments, where asterisks and number signs refer to the statistically significant difference of means compared to the untreated vehicle control and the pairwise comparison, respectively (*p* < 0.05).

**FIGURE 7:**
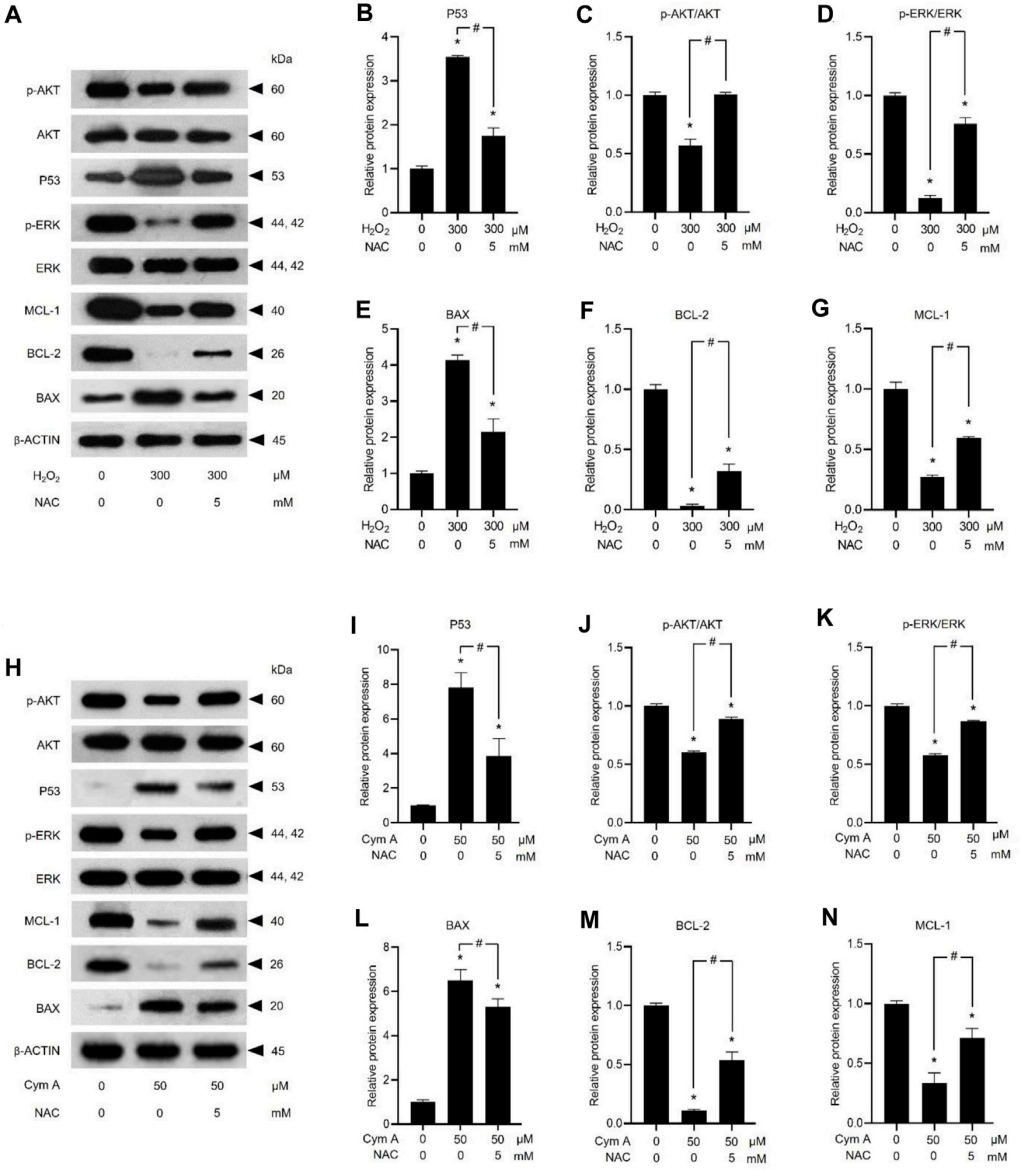
Cymensifin A (Cym A) upregulates tumor suppressor protein (P53) and modulates apoptosis/survival-associated proteins in lung cancer cells via the reactive oxygen species (ROS)-mediated pathways. H460 cells were used in this experiment. Cells exposed to 0 [vehicle control, using 0.5% (v/v) dimethyl sulfoxide] or 50 μM Cym A or 300 μM hydrogen peroxide (H_2_O_2_) (positive control) for 24 h were assessed by a Western blot analysis (**A–N**, original blots can be found in Supplementary Material), where P53 **(A,B,H,I)**, and other apoptosis/survival-associated proteins, including AKT **(A,C,H,J)**, ERK **(A,D,H,K)**, BAX **(A,E,H,L)**, BCL-2 **(A,F,H,M)**, and MCL-1 **(A,G,H,N)** were analyzed (prefix p for AKT and ERK refers to their phosphorylated portion). The β-ACTIN served as a reference protein. Cells pretreated with 5 mM *N*-acetylcysteine (NAC) (ROS inhibitor) for 30 min before exposure to Cym A or H_2_O_2_ served as negative controls. The numerical data represent means ± standard deviations derived from three independent experiments, where asterisks and number signs refer to the statistically significant difference of means compared to the untreated vehicle control and the pairwise comparison, respectively (*p* < 0.05).

**FIGURE 8:**
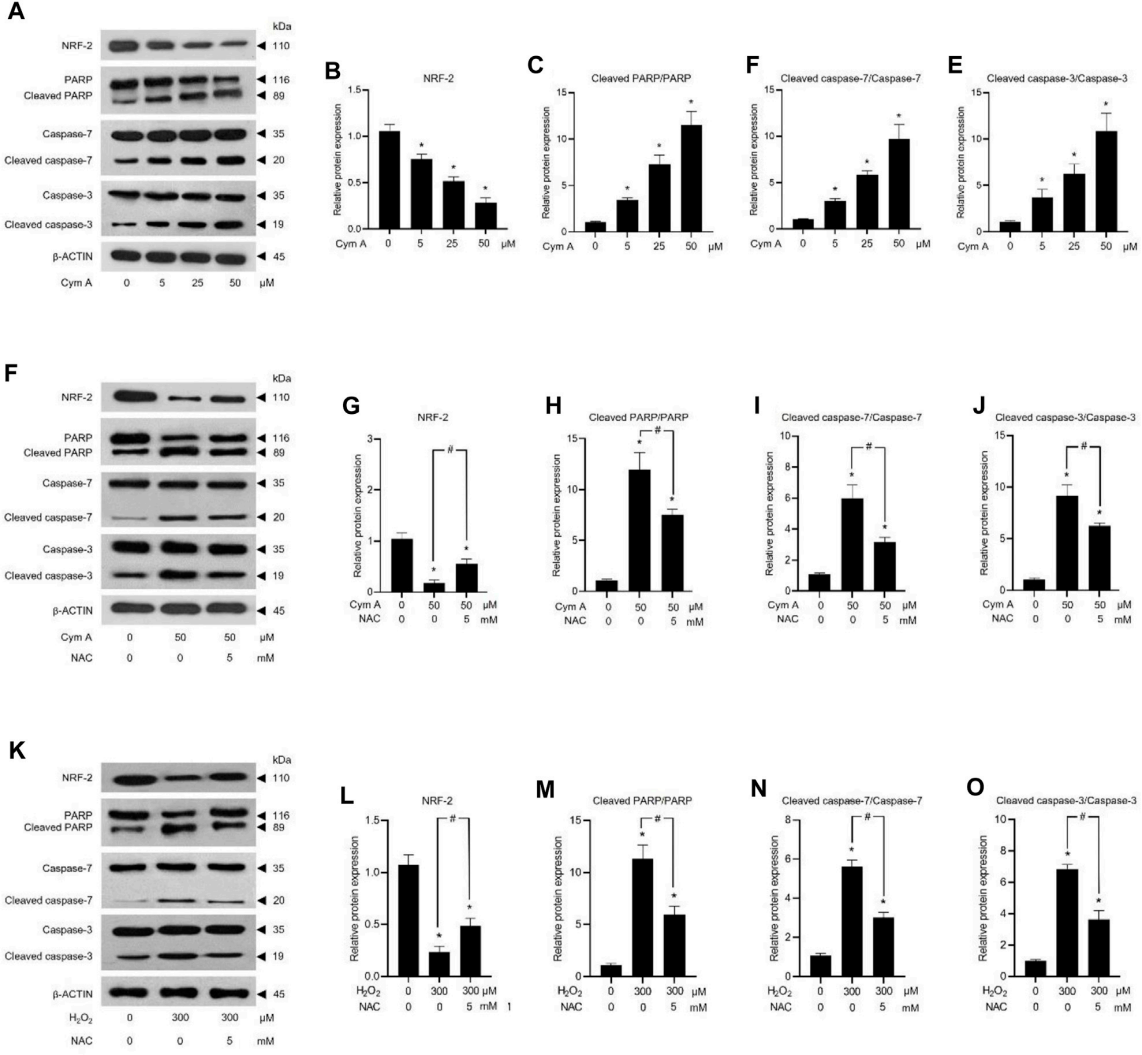
Cymensifin A (Cym A) causes lung cancer cell death via reactive oxygen species (ROS)-mediated mitochondrial outer membrane permeabilization (MOMP) and DNA damage. H460 cells were used in this experiment. Cells exposed to varying concentrations [0 (vehicle control, using 0.5% (v/v) dimethyl sulfoxide], 5, 25, or 50 μM) of Cym A or 300 μM hydrogen peroxide (H_2_O_2_) (positive control) for 24 h were assessed by a Western blot analysis (**A–O**, original blots can be found in Supplementary Material), where nuclear factor erythroid 2–related factor 2 (NRF-2)–a ROS-resistant regulator **(A,B,F,G,K,L)**, poly (ADP-ribose) polymerase (PARP) and its cleaved segments **(A,C,F,H,K,M)**, caspase 3 and its cleaved segments **(A,E,F,J,K,O)**, and caspase 7 and its cleaved segments **(A,D,F,I,K,N)**, were analyzed. The β-ACTIN served as a reference protein. Cells pretreated with 5 mM *N*-acetylcysteine (NAC) (ROS inhibitor) for 30 min before exposure to Cym A or H_2_O_2_ served as negative controls. The numerical data represent means ± standard deviations derived from three independent experiments, where asterisks and number signs refer to the statistically significant difference of means compared to the untreated vehicle control and the pairwise comparison, respectively (*p* < 0.05).

We established a set of experiments using H_2_O_2_ (as an exogenous ROS) and NAC (a ROS inhibitor) to verify that the central mechanism underlying the anticancer activity of Cym A was mediated by ROS pathways (Figures 6–8). Cym A at 50 μM caused H460 cell death (∼40% of the vehicle control cells) after exposure for 24 h, and this apoptotic role of the compound was fully suppressed by a 30 min pretreatment of cells with 5 mM NAC (Figure 6A). These findings correlated to their qualitative results derived from a nuclear staining assay (Figure 6B), where the number of dead cells decreased in the conditions pretreated with 5 mM NAC. A similar trend of results was observed with the tests using 300 μM H_2_O_2_ instead of 50 μM Cym A, although H460 cells were more susceptible to this ROS than Cym A (Figure 6A).

ROS-mediated cancer cell death caused by 50 μM Cym A was proved in comparison with 300 μM H_2_O_2_ to upregulate P53 (Figures 7A, B, H, I) and BAX (Figures 7A, E, H, L), deactivate AKT (Figures 7A, C, H, J) and ERK (Figures 7A, D, H, K), and downregulate BCL-2 (Figures 7A, F, H, M) and MCL-1 (Figures 7A, G, H, N). The pretreated cells with 5 mM NAC for 30 min could suppress all these ROS effects, either from Cym A or H_2_O_2_. Interestingly, unlike 300 μM H_2_O_2_, Cym A at 50 μM strongly upregulated P53 and BAX (Figures 7H, I, L), up to 2-fold higher than 300 μM H_2_O_2_ (Figures 7A, B, E), even with or without the pretreatment with NAC. Another study to unveil the roles of Cym A in ROS-mediated apoptosis via its link with MOMP and DNA damage was conducted (Figure 8). Cym A could decrease the expression of NRF-2 (a regulator in cellular ROS resistance) (Figures 8A, B) but increase the cleavage of caspase 3 (Figures 8A, E), caspase 7 (Figures 8A, D), and PARP (Figures 8A, C) in dose-dependent manure. In confirming that Cym A acted as a ROS (Figures 8F–O), it was explicit that Cym A and H_2_O_2_ exerted similar oxidant activity in modulating these proteins, and such activity could be suppressed by ROS inhibitor (NAC).

## 4 Discussion

Chemotherapy is a fundamental treatment that potentially increases the survival rate of lung cancer patients. Unfortunately, the susceptibility to anticancer drugs usually decreases when tumor cells acquire drug resistance after the initial administration (Cohen and Lippard, 2001). The overexpression of defensive mechanisms to prevent cellular stress and damage is a strategy of lung cancer cells to resist chemotherapy (Housman et al., 2014; Bukowski et al., 2020). Oxidative stress caused by chemotherapeutic drugs is normally managed at the lethal level in cancer cells through the upregulated expression of cellular antioxidants (Yang et al., 2018; van Loenhout et al., 2020). Malignant cells have a high level of ROS but can maintain a redox balance due to their strong antioxidant capacity (van Loenhout et al., 2020). Hence, novel anticancer agents that enhance the role of ROS in cancer cell death and parallelly suppress cellular antioxidants would potentially be promising candidates to defeat drug resistance in lung cancer patients.

In this study, the anticancer mechanism of Cym A has started to be elucidated. Its cytotoxicity against lung cancer cells is comparable to cisplatin, a prevalent anticancer drug for lung cancer therapy. Our findings demonstrated that Cym A acts like ROS (H_2_O_2_), causing lung cancer cell death. The mitochondrial ROS overproduction leading to MOMP-mediated apoptosis is the principal mode of action of many anticancer drugs (Yang et al., 2018). Along with the elevated mitochondrial ROS production, the inhibition of cellular antioxidants often coexists. Thus, downregulation of cellular ROS can play a significant role in multidrug resistance in cancer cells (Wang et al., 2018). Cym A behaves like ROS and significantly downregulates cellular antioxidants, including catalase, SOD1, and TRX. In addition, Cym A acts as a suppressor of NRF-2, a regulator in cellular resistance to ROS. The suppressed anticancer activity of Cym A due to the pretreatment with an antioxidant (NAC) has also confirmed that Cym A induces cancer cell death via ROS-mediated pathways.

Various stressors, including ROS, can trigger apoptosis via MOMP. Mitochondrial membrane integrity relies mainly on BCL-2 family proteins, which can be intervened by an excess amount of cellular ROS (Trachootham et al., 2009). Whether a cell will undergo apoptosis or not is often determined by the balance of these BCL-2 family proteins, including pro-apoptotic (e.g., BAX) and pro-survival (e.g., BCL-2 and MCL-1) ones (Hemann and Lowe, 2006; Bukowski et al., 2020; Strasser and Vaux, 2020). Overexpressed BCL-2 and MCL-1 have been recognized for various types of lung cancer cells (Porebska et al., 2006; Xiang et al., 2018). Cym A does not only downregulate BCL-2 and MCL-1 but also upregulates BAX significantly, leading to the provoked MOMP-mediated cell death. Other cell survival-associated proteins, such as AKT and ERK, have shown their close link to tumorigenesis and lung cancer progression (Cao et al., 2019; Tan, 2020). Activated AKT via phosphorylation in cancer cells contributes to their survival, allowing them to withstand various cell death stimuli (Tan, 2020). While phosphorylated ERK mainly controls cell growth, proliferation, and differentiation, the overactivation of this protein has been found typically in up to 90% of lung cancers (Cao et al., 2019). The deactivation of AKT and ERK in lung cancer cells is also observed when treating cells with Cym A for 24 h.

P53 is a tumor suppressor and transcription factor, playing crucial roles in monitoring cell homeostasis, guiding cell cycle arrest, and triggering apoptosis (Yang et al., 2018). The expression of BCL-2 family proteins is also governed by P53 (Vazquez et al., 2008). Moreover, P53 modulates the machinery of the PI3K/AKT/mTOR signaling pathway to control a broad array of cellular processes, including tumorigenesis, proliferation, and apoptosis (Tan, 2020). Studies have shown that chemotherapies efficiently deplete AKT and ERK phosphorylation but overexpress P53 to induce cancer cell apoptosis (Chai et al., 2010; Roy Choudhury et al., 2010). Restraining or losing P53 has been found in aggressive lung cancer cells, while P53 mutation in these cells results in poor response to radiation and cisplatin treatment (Viktorsson et al., 2005). The shifted expression of BCL-2 family proteins and the deactivation of AKT and ERK signals by Cym A may be the consequences of P53 activation. However, we found that Cym A could exert its cytotoxic effects on lung cancer cells regardless of their genetic variation in the *TP53* gene, as H23 cells, the *TP53* mutants, were more susceptible to Cym A than other lung cancer cells harboring *TP53* wild type (i.e., A549, H292, and H460). These results would support that P53 might not be the central mechanism underlying lung cancer cell apoptosis caused by Cym A. Additional studies to unveil how Cym A overcomes this aggressive feature (*TP53* mutation) of lung cancer cells would expand its pharmaceutical potential in lung cancer therapy.

The downstream events of MOMP are typically led by the release of cytochrome *c* and a series of caspase activations. The cleavage of execution caspases 3 and 7 is the way to activate their apoptotic functions. These activated caspases can further cleave PARP, contributing to genome instability, DNA damage, and apoptosis (Curtin and Szabo, 2020). Our findings demonstrate that Cym A promotes the cleavage of caspase 3/7 and PARP, and this role of Cym A has a strong link with ROS-mediated cell death. These results are consistent with what was observed in the nuclear staining assay, where we can find an increased number of dead cells with condensed and fragmented nuclei after treatments with increased concentrations of Cym A. We conclude that Cym A induces lung cancer cell death via ROS-mediated MOMP and DNA damage. Its molecular mechanisms underlying cancer cell apoptosis may include but are not limited to provoking cellular ROS stress, suppressing cellular antioxidants (catalase, SOD1, and TRX) and ROS-resistant regulator (NRF-2), upregulating the tumor suppressor (P53) and pro-apoptotic protein (BAX) but downregulating pro-survival proteins (BCL-2 and MCL-1) and deactivating survival signals (AKT and ERK), and increasing apoptotic enzyme cleavages (caspase 3/7 and PARP). This phytochemical shows effective anticancer activity and would be a promising pharmaceutical candidate for the next-generation of lung cancer therapy.

## Data Availability

The original contributions presented in the study are included in the article/Supplementary Material, further inquiries can be directed to the corresponding author.
