# Supplementary material for: Cymensifin A: a promising pharmaceutical candidate to defeat lung cancer via cellular reactive oxygen species-mediated apoptosis
Source: Front Pharmacol. 2024 Apr 11;15:1361085. doi: 10.3389/fphar.2024.1361085 (PMC11043475; doi:10.3389/fphar.2024.1361085)

Supplementary Material

Cymensifin A: A promising pharmaceutical candidate to defeat lung cancer via cellular reactive oxygen species-mediated apoptosis

Bruno Cesar Costa Soares [1,2], Hnin Ei Ei Khine [2], Boonchoo Sritularak [3,4], Pithi Chanvorachote [5,6], Rosa Alduina [7], Rungroch Sungthong [2], Chatchai Chaotham [2,6,*]

[1] Pharmaceutical Sciences and Technology Program, Faculty of Pharmaceutical Sciences, Chulalongkorn University, Bangkok 10330, Thailand

[2] Department of Biochemistry and Microbiology, Faculty of Pharmaceutical Sciences, Chulalongkorn University, Bangkok 10330, Thailand

[3] Department of Pharmacognosy and Pharmaceutical Botany, Faculty of Pharmaceutical Sciences, Chulalongkorn University, Bangkok 10330, Thailand

[4] Center of Excellence in Natural Products for Ageing and Chronic Diseases, Faculty of Pharmaceutical Sciences, Chulalongkorn University, Bangkok 10330, Thailand

[5] Department of Pharmacology and Physiology, Faculty of Pharmaceutical Sciences, Chulalongkorn University, Bangkok 10330, Thailand

[6] Center of Excellence in Cancer Cell and Molecular Biology, Faculty of Pharmaceutical Sciences, Chulalongkorn University, Bangkok 10330, Thailand

[7] Department of Biological, Chemical and Pharmaceutical Sciences and Technologies (STEBICEF), University of Palermo, Palermo 90128, Italy

* Corresponding author: Chatchai Chaotham (e-mail: cchoatham@gmail.com)

Original western blots for Figure 4

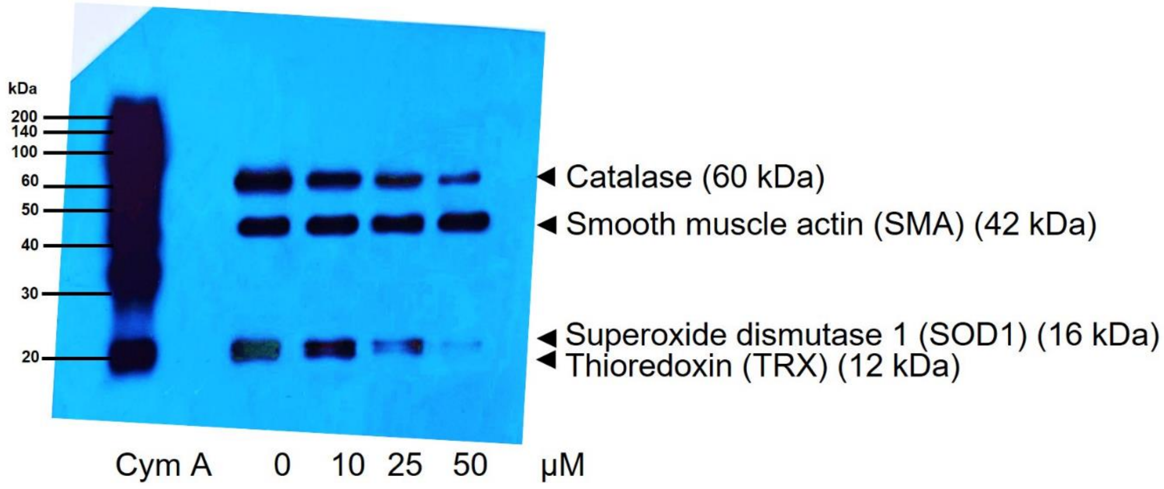

Original western blots for Figure 5

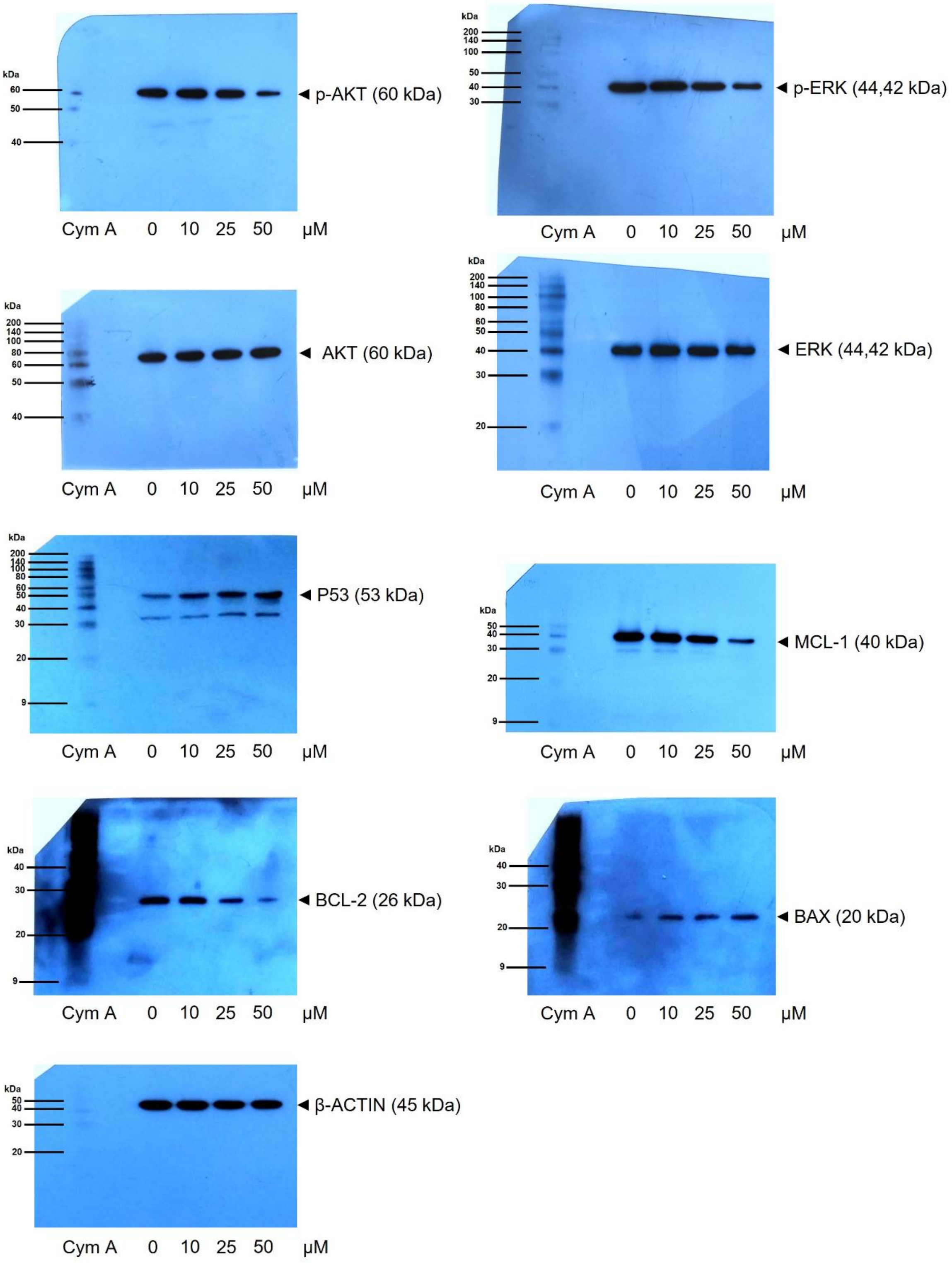

Original western blots for Figure 7 (a-g)

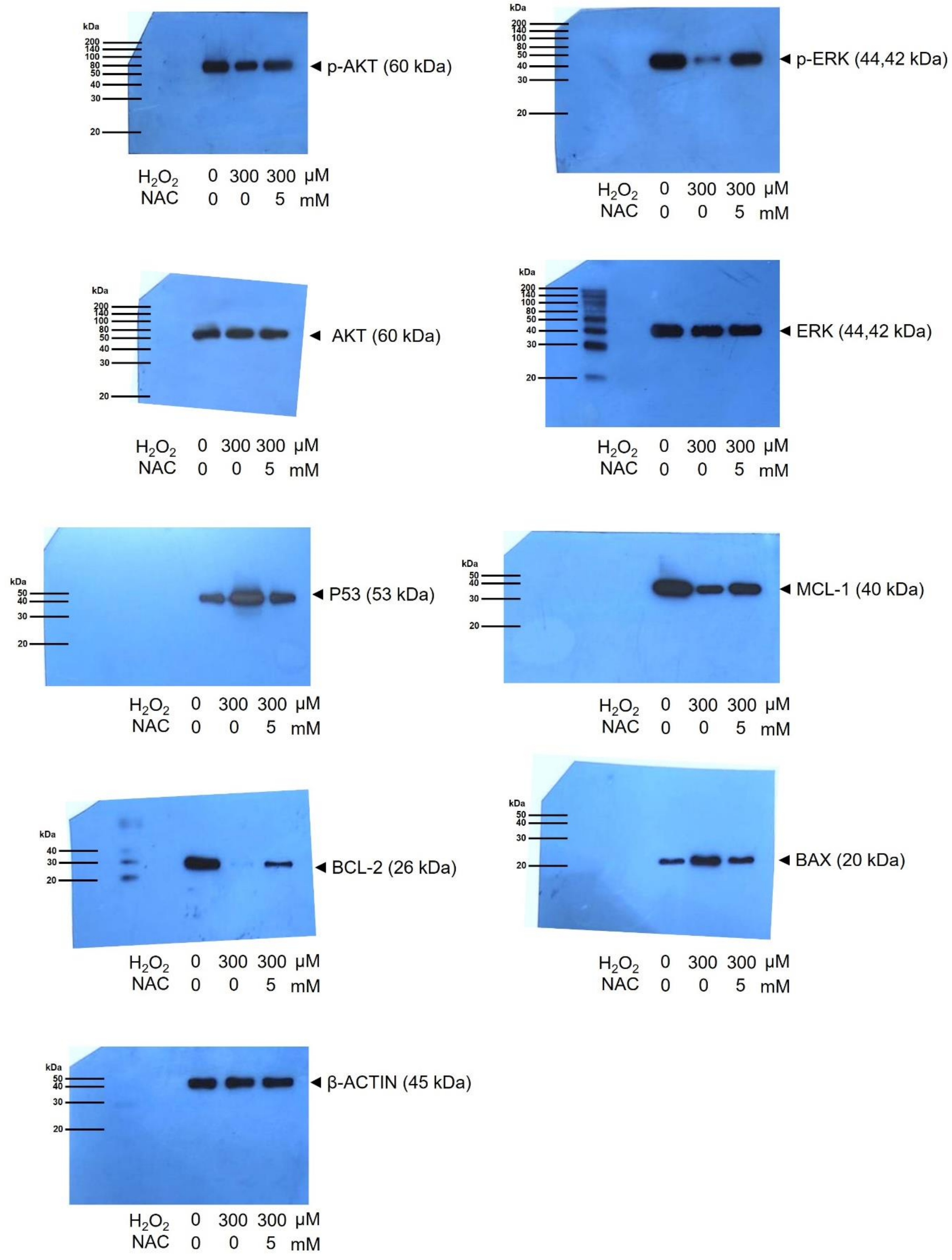

Original western blots for Figure 7 (h-n)

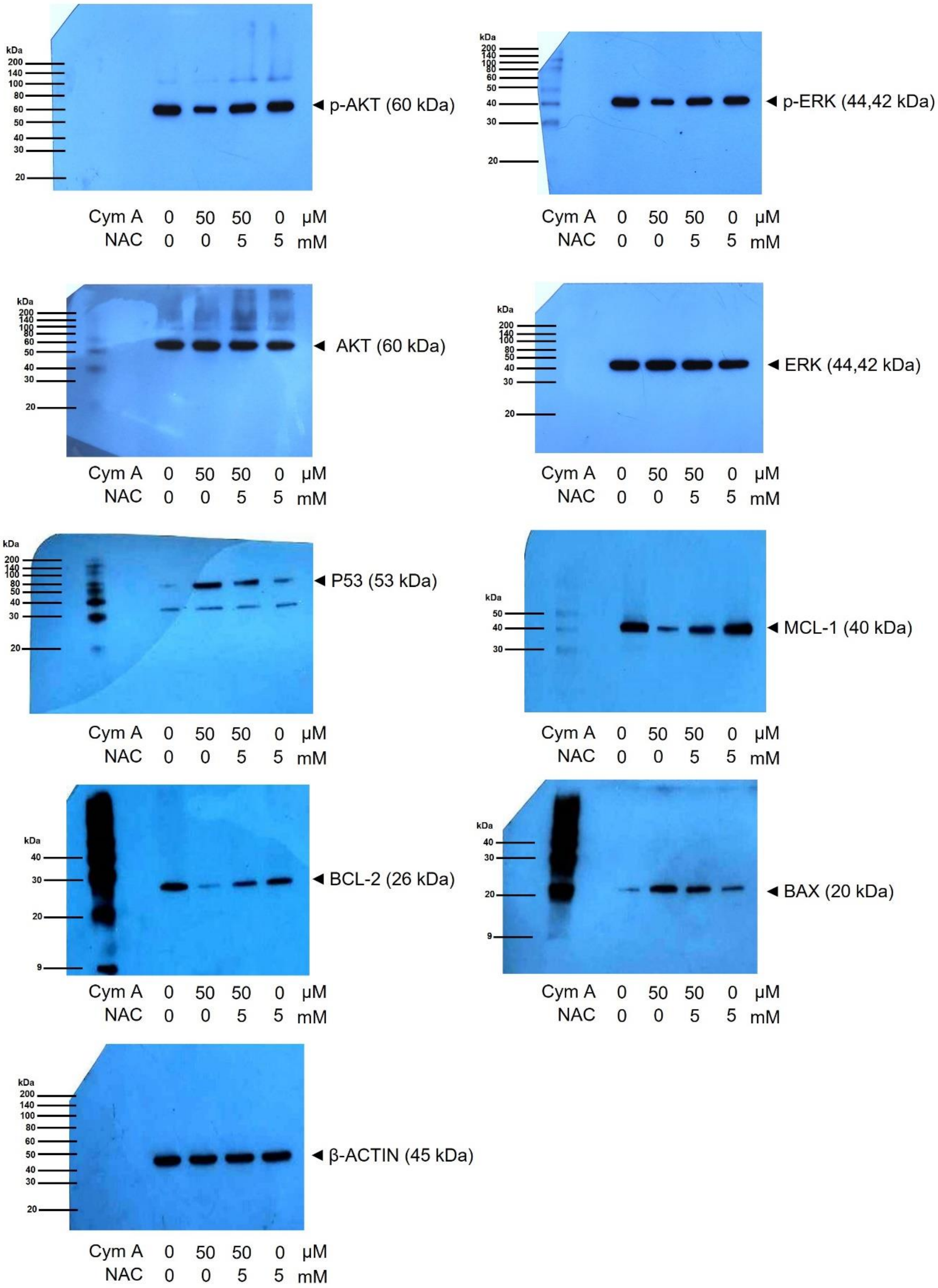

Original western blots for Figure 8

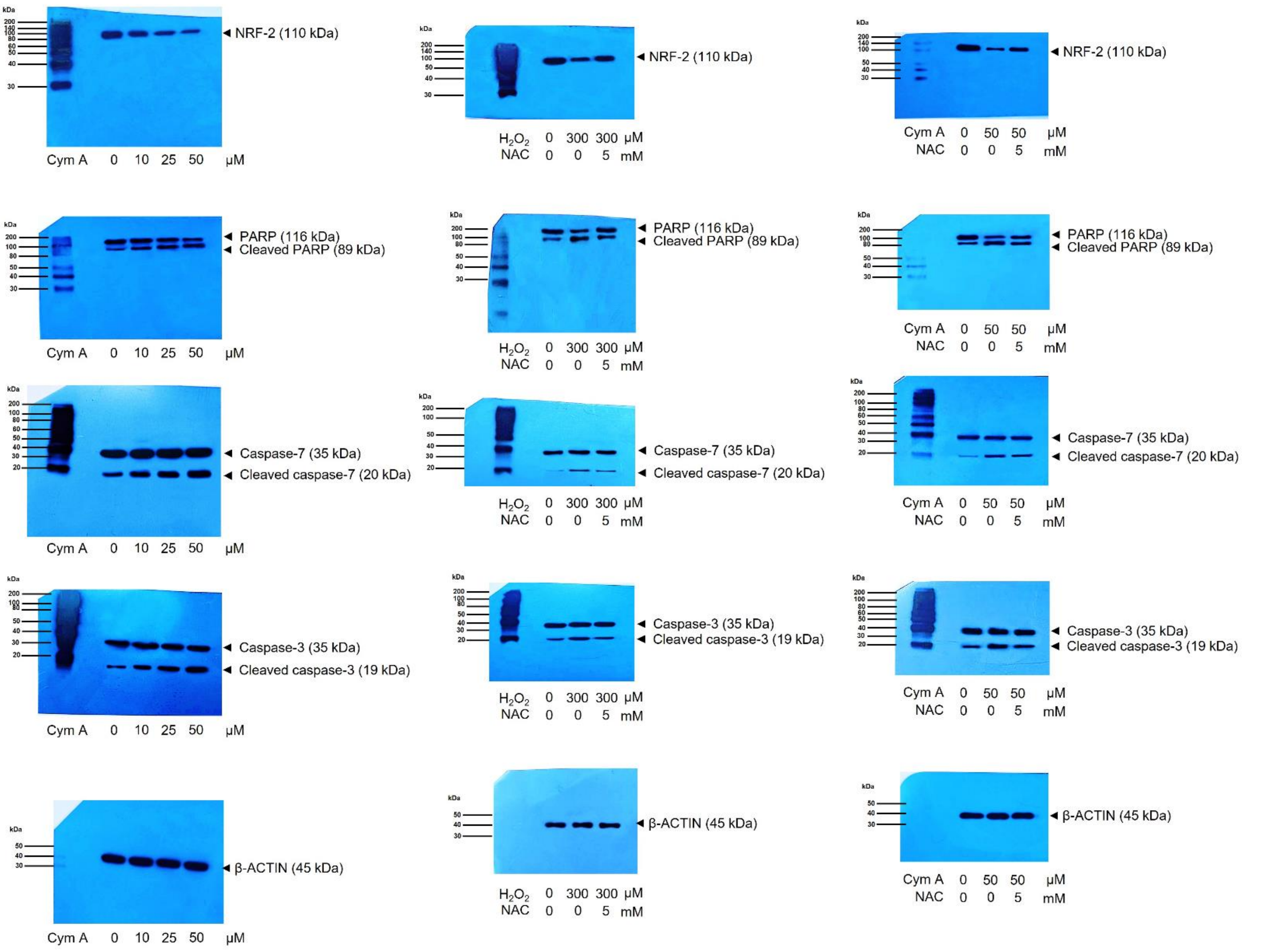

Supplement: Supplementary file 1 [file Image1.pdf]
